# Using a Somatosensory Controller to Assess Body Size for Size-Specific Dose Estimates in Computed Tomography

**DOI:** 10.1155/2018/2734297

**Published:** 2018-05-31

**Authors:** Jay Wu, Ruo-Ping Han, Yan-Lin Liu

**Affiliations:** ^1^Department of Biomedical Imaging and Radiological Sciences, National Yang-Ming University, Taipei, Taiwan; ^2^Department of Management Information Systems, Central Taiwan University of Science and Technology, Taichung, Taiwan; ^3^Institute of Nuclear Engineering and Science, National Tsing Hua University, Hsinchu, Taiwan

## Abstract

Computed tomography (CT) has been widely used in the healthcare environment. Presently, the radiation dose in CT is determined using the size-specific dose estimate (SSDE). Accurate assessment of individual's body size is essential for dose estimation. In this study, we integrated a somatosensory controller with a CT scanner to measure patient's anterior-posterior diameter (APD) and lateral diameter (LATD) and calculate the corresponding effective diameter (ED). A total of 108 individuals with an average age of 38.6 years were enrolled in this study. Microsoft Kinect was used to acquire the depth image of subjects. A grayscale-to-surface height conversion curve was created using acrylic sheets for APD estimation. The APD, LATD, and ED were measured and compared with the results obtained using F ruler and CT images. The mean absolute differences for APD, LATD, and ED between Kinect and F ruler measurements were 5.2%, 1.3%, and 2.5%, respectively, while those between Kinect and CT measurements were 8.8%, 2.6%, and 5.0%, respectively. Kinect can replace CT or F ruler for real-time body size measurements. The use of the somatosensory controller has the advantages of simple, low cost, no radiation, and automatic calculation. It can accurately estimate patient's APD, LATD, and ED for SSDE.

## 1. Introduction

Computed tomography (CT) accounts for approximately half of the collective dose for medical exposure, and the number of CT scans increases by 10% to 15% annually [1]. Therefore, radiation doses in CT scans should be accurately estimated. The existing dose estimation methods include the multiple scan average dose (MSAD) and the CT dose index (CTDI). These methods consider only scanning regions and scanner outputs. American Association of Physicists in Medicine (AAPM) proposed the size-specific dose estimate (SSDE) to account for the body size of individuals [2, 3]. The anterior-posterior distance (APD) and the lateral distance (LATD) are measured from the axial CT image of subjects and converted to the effective diameter (ED) which pinpoints the dose-conversion factor for the correction of volumetric CTDI (CTDI_vol_). Hence, obtaining information about body size is important for estimation of patient doses in CT.

The water-equivalent diameter (*D*_*w*_) [4, 5], which is the diameter of a circular water phantom with an equivalent cross sectional area to that in the CT image, was proposed as another body size index. An anterior-posterior topogram is required for converting the X-ray attenuation information of the scanning region to *D*_*w*_. This method has certain accuracy; however, the attenuation due to the scanning couch may significantly increase the *D*_*w*_ [6]. In addition, this method can only be performed after X-ray exposure. Cook et al. [7] recorded subject's body size in the standing position using a somatosensory controller. The estimates are prone to relatively large variations because of the differences in body postures and the looseness of clothes.

Factors such as patient's age, height, weight, and abdominal circumference can be related to ED [2, 8–10]. The age-based abdominal ED estimation exhibited relatively large errors, especially for children [11]; up to 44% difference in ED was found for teenagers [12]. APD plus LATD was also used to pinpoint the corresponding conversion factor for correction of CTDI_vol_ [13]. Since measuring the dimensions of body size is a laborious task, an accurate and automatic method for estimating ED should be investigated. In this study, we integrated a somatosensory controller with a CT scanner to accurately measure APD and LATD and further calculate ED in real time for size-specific dose estimates in CT.

## 2. Materials and Methods

### 2.1. Subjects

A total of 108 individuals (44 males and 64 females) with an average age of 38.6 years (24–82 years), average height of 164.2 cm (154–185 cm), and average weight of 62.4 kg (43–83 kg) were enrolled in this study. Their signed informed consent was obtained. All experimental protocols were approved by the Research Ethics Committee of China Medical University and Hospital. The methods were carried out in accordance with ICH-GCP guidelines.

### 2.2. Image Acquisition

**Figure 1:**
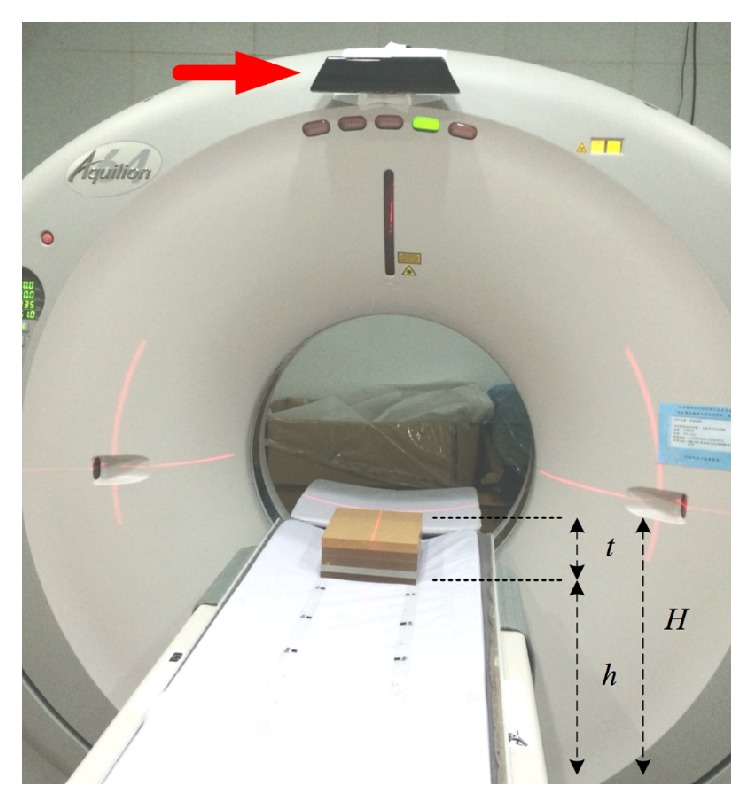
Kinect (arrow) was installed on the CT frame. The RGB camera and the infrared transmitter and receiver were faced perpendicular to the examination couch. *h* is the couch height, *t* is the phantom thickness, and *H* represents the surface height.

A somatosensory controller, Microsoft Kinect (Microsoft, USA), was fixed at the upper edge of a 64-slice CT scanner (Toshiba Medical Systems Corporation, Japan) to acquire depth images of subjects (Figure 1). The Kinect comprises three lenses: a red-green-blue (RGB) camera, an infrared transmitter, and an infrared receiver. The operating range is between 0.5 m and 5.0 m. The infrared transmitter emits a reference speckle pattern of structured lights onto the scene. The projected pattern is then captured by the infrared receiver from an offset point of view. The disparity between the projected and the reference patterns can be converted to depth information through a triangulation process. The grayscale value in the depth image indicates the relative distance from the reflector to the Kinect sensor. The depth resolution is approximately 2 mm within 1-m range. Detailed information about the mathematical model of depth measurements of Kinect can be found elsewhere [14]. The Windows SDK 1.8 toolbox for Kinect was used for image acquisition and further automatic image processing.

### 2.3. Body Size Estimation

**Figure 2:**
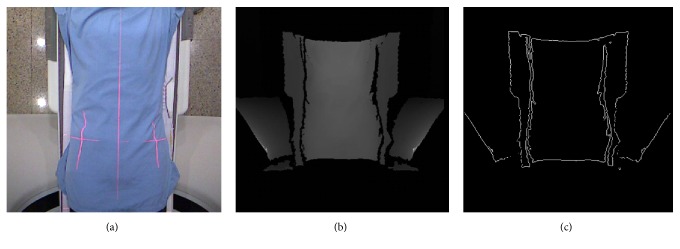
Images acquired by (a) the RGB camera and (b) the infrared receiver of Kinect and (c) filtered with the Prewitt operator.

For body width measurements, the depth image was filtered with the Prewitt operator for edge detection (Figure 2). Seeds were placed along the central axis of the edge enhanced image and moved horizontally to both sides until reaching the inner edges of the body. The minimum length between the inner edges along the body axis was automatically searched and taken as the body width. The LATD was further calculated as follows:

(1) $$\begin{array}{c} LATD=N_{W}\times \text{Pixel Size}, \end{array}$$

where *N*_*W*_ is the pixel number of body width. The pixel size can be calculated in advance by dividing the width of the examination couch by the pixel number of the couch.

A calibration procedure was performed for body thickness estimation. The couch height (*h*) was fixed at 91 cm. 2-cm thick acrylic sheets were placed on the couch and stacked to produce the phantom thickness (*t*) of 10 to 36 cm, leading to the surface height (*H*), from the floor to the phantom surface, ranging from 101 to 127 cm (Figure 1). For each surface height, the depth image was acquired and a 10 × 10 region of interest (ROI) was drawn in the center of the phantom. The grayscale-to-surface height conversion curve was established.

For body thickness measurements, a 10 × 10 ROI was drawn in the central axis of subject's depth image corresponding to the location of body width estimation. The mean grayscale value was converted to the surface height according to the above conversion curve. The APD was then calculated by subtracting the recorded couch height from the surface height. After acquiring subject's LATD and APD, ED was calculated as follows:

(2) $$\begin{array}{c} ED=\sqrt{LATD\times APD}. \end{array}$$

### 2.4. Evaluation of Examination Gown Colors

Errors in the depth image mainly come from the characteristics of reflectors. Since different colors of examination gowns were used in hospitals, the effect of gown colors on APD measurements was evaluated. The white, green, red, and blue gowns were placed on a 15-cm thick acrylic phantom, respectively. The depth image was captured and a 10 × 10 ROI was placed at the center of the gown. The APD was calculated based on the grayscale-to-surface height conversion curve.

### 2.5. Body Size Validation

Subject's body size obtained by the somatosensory controller was validated. Manual measurements of APD and LATD using the *F* ruler, a kind of caliper, were performed on 88 subjects at two fingers above the iliac crest. Additionally, CT scans were performed on 20 patients; APD and LATD were manually measured from the CT images by a radiologist with more than 10 years of experience. The mean absolute difference (MAD) was calculated for comparison between methods as follows:

(3) $$\begin{array}{c} \text{MAD}=\frac{1}{N}\overset{N}{\underset{n=1}{\sum }}\frac{\left|x_{n}-y_{n}\right|}{y_{n}}, \end{array}$$

where *x* is the result measured by Kinect and *y* is the one measured by the *F* ruler or CT image. *N* is the number of subjects.

### 2.6. Statistical Methods

In order to assess the adequacy of replacing *F* ruler and CT measurements by Kinect measurements, the simple linear regression model was fitted where the Kinect measurements are the dependent variable and the other two measurements are the independent variables. The hypothesis tests are focused on whether the intercept (*β*_0_) and slope (*β*_1_) of the true regression line are zero and one, respectively. If the above statement is true, meaning that given a value of *F* ruler or CT, the averaged Kinect measurement is exact to the given value of *F* ruler or CT, i.e., *E*[*K*∣*F*] = *F* and *E*[*K*∣CT] = CT. In such case, we can replace *F* ruler and CT results by Kinect results. The Bonferroni correction [15] was applied to adjust the significant level of each separate test to 0.025, since *H*_0_ : *β*_0_ = 0 and *H*_0_ : *β*_1_ = 1 were tested simultaneously. The statistical analysis was carried out by SPSS version 19.0.

## 3. Results

**Figure 3:**
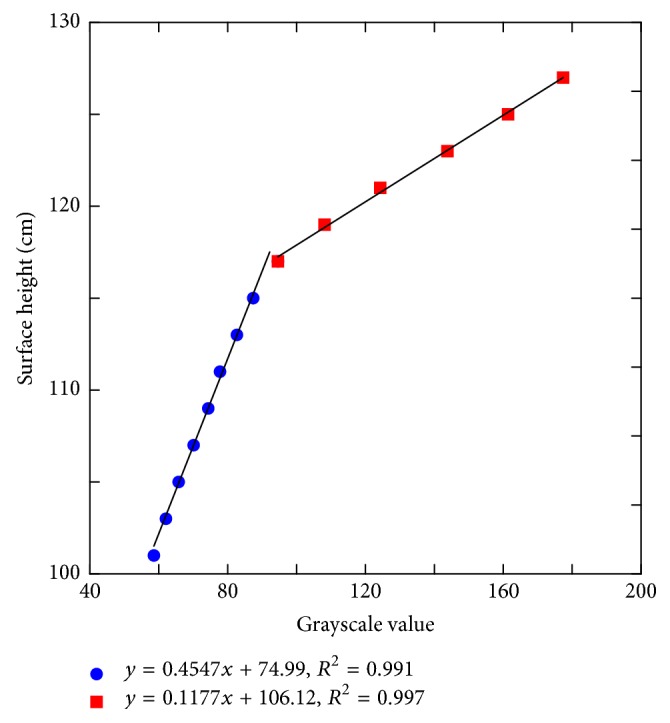
Grayscale-to-surface height conversion curve for APD estimation. Piecewise linear fitting was performed using the grayscale value of 92.2 as a breakpoint. The *R*^2^ values of the fitting results were higher than 0.990.

Figure 3 shows the grayscale-to-surface height conversion curve according to the Kinect scans of acrylic sheets. The data were divided into two groups by the grayscale value of 92.2 or by the corresponding surface height of 117 cm. Piecewise linear fitting was conducted to establish the following relationships:

(4) y=0.4547x+74.99if  x<92.2,y=0.1177x+106.12if  x≥92.2,

where *x* is the grayscale value and *y* is the surface height, the distance from the floor to the body surface in cm. The *R*^2^ values of the two equations were higher than 0.990, indicating satisfactory fitting results. The APD was calculated by subtracting the recorded couch height from the surface height.

**Figure 4:**
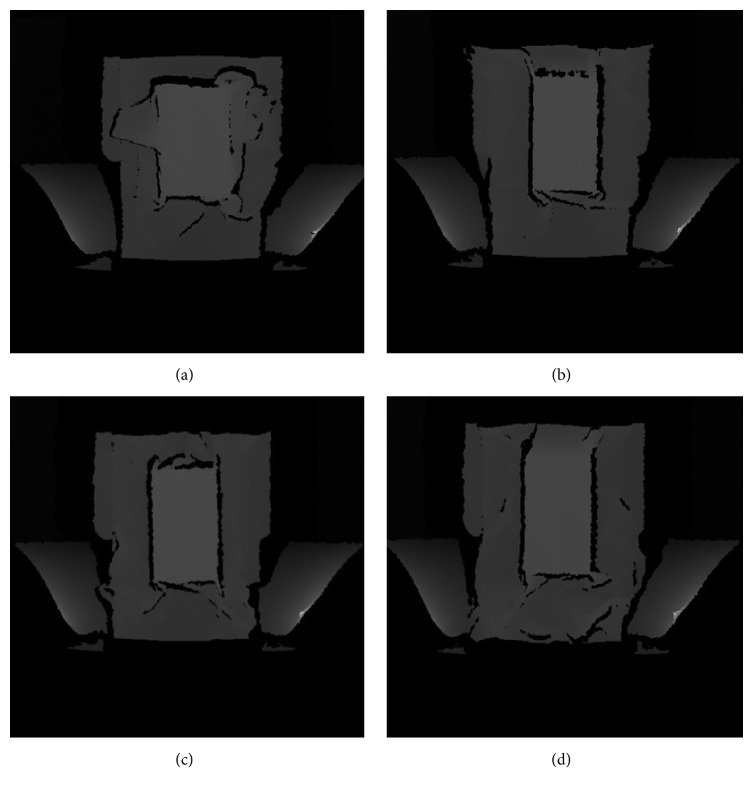
Depth images of the examination gowns. The (a) white, (b) green, (c) red, and (d) blue gowns were placed on top of a 15-cm thick acrylic phantom, respectively. Parts of the white and blue gowns were deliberately spread on the couch.

Figure 4 shows the depth images of different colors of gowns. The mean grayscale intensities under the white, green, red, and blue gowns were 68.50, 69.71, 68.44, and 69.76, respectively. The corresponding surface heights were 106.14, 106.69, 106.11, and 106.71 cm, and the APD results were 15.14, 15.69, 15.11, and 15.71 cm. The standard error of APD was 3.3 mm, which is slightly higher than the depth resolution of Kinect. Hence, the color of examination gowns does not markedly influence the APD estimation.

**Figure 5:**
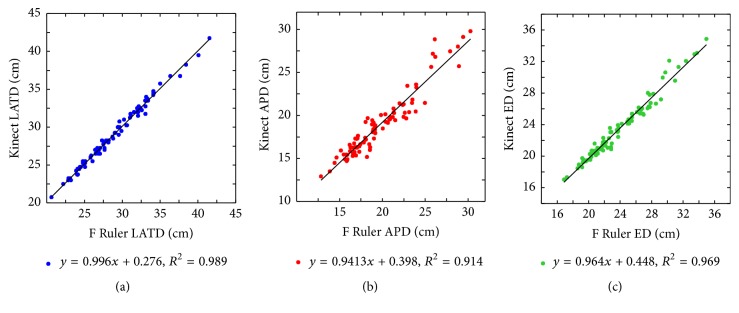
Scatter plots of Kinect versus *F* ruler measurements. The (a) LATD, (b) APD, and (c) ED of the two methods matched very well. The *R*^2^ values of linear fitting were all larger than 0.910.

Table 1 shows the comparison of *F* ruler and Kinect measurements. The mean LATD, APD, and ED results of *F* ruler were 28.99, 19.70, and 23.88 cm, respectively, and the corresponding values of Kinect were 29.14, 18.94, and 23.46 cm, respectively. Among the subjects, the MAD for LATD, APD, and ED between Kinect and *F* ruler was 1.3%, 5.2%, and 2.5%, respectively.  Figure 5 illustrates the scatter distributions between the Kinect and *F* ruler measurements. The Kinect results were highly consistent with the *F* ruler results. The correlation coefficients of linear fitting for the three diameters were all larger than 0.910.  Table 2 shows the *p* values on testing *H*_0_ : *β*_1_ = 1 and *H*_0_ : *β*_0_ = 0. The *p* values were all larger than 0.025, indicating that *E*[*K*∣*F*] = *F* holds. Therefore, the Kinect measurements are equivalent to the *F*-ruler measurements.

**Figure 6:**
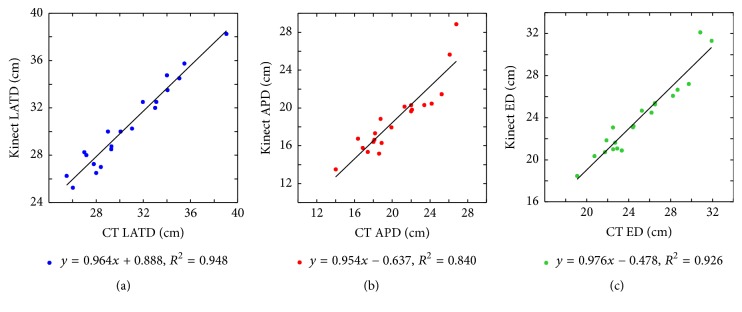
Scatter plots of Kinect versus CT measurements. The (a) LATD, (b) APD, and (c) ED of the two methods matched very well. The *R*^2^ values for LATD and ED were larger than 0.920, and the *R*^2^ value for APD was 0.840.

Table 3 shows the comparison of CT and Kinect measurements. The mean LATD, APD, and ED values measured using CT images were 30.71, 20.41, and 25.01 cm, respectively, whereas the results measured using Kinect were 30.49, 18.83, and 23.93 cm, respectively. The Kinect measurements were slightly less than the CT results. The MAD for LATD, APD, and ED was 2.6%, 8.8%, and 5.0%, respectively.  Figure 6 shows the correlation between Kinect and CT measurements for LATD, APD, and ED. The *R*^2^ values for LATD and ED were larger than 0.920, whereas the *R*^2^ value for APD decreased to 0.840.  Table 4 shows the *p* values on testing *H*_0_ : *β*_1_ = 1 and *H*_0_ : *β*_0_ = 0. The *p* values were all larger than 0.025, meaning that *E*[*K*∣CT] = CT holds. Therefore, the Kinect measurements are equivalent to the CT measurements.

## 4. Discussions

Kinect measurements are robust in the indoor environment as long as the distance between the Kinect sensor and object is in the operating range between 5 cm to 5 m and 57° horizontal field of view. In addition, the measurements are not sensitive to patient's posture and orientation. The body size indices obtained using Kinect match very well with those using the *F* ruler. The major source of error in LATD is the looseness of examination gowns, causing a slightly larger result of Kinect. This effect can also be observed in Figure 4(a), where the white gown was spread on the couch deliberately. The error in LATD measurements can be avoided if technicians pay a little attention and carefully stuff the gown below the patient while positioning the patient. A slightly lower mean APD result was achieved by Kinect. This could be due to no breathing control applied during the depth image acquisition.

The mean results of CT were slightly higher than those of Kinect, especially in APD. This is mainly because the patients were in the full inhalation phase during CT scanning, whereas they were breathing freely during Kinect acquisition. Breathing is also the reason that the MAD of APD was larger than that of LATD. If we control subject's breathing for Kinect scans, the MAD of APD should be effectively reduced. However, a prolonged breath hold time or a secondary breath hold may cause patients to be uncomfortable and unable to remain still during CT scanning.

The somatosensory controller is easy to implement and can provide real-time body size information for SSDE calculation. In this study, APD is calculated by subtracting the table height from the surface height. The table height is a variable for each patient and was extracted from the DICOM header of CT images. The surface height was obtained from the grayscale-to-surface height conversion curve. Since the conversion curve is independent to CT machines, it only needs to be created once for later use. From Figure 2(b), we can also observe that the resolution in APD is enough to reflect the continuous change of body thickness.

There are two methods for SSDE corrections [16]. When the mean SSDE is averaged from the SSDE values along the *Z* axis of the scanning range, two markers should be placed next to the subject to correlate the scanning range with the *Z* position. Multiple LATD and APD can be measured along the *Z* axis of the depth image. On the other hand, when the mean SSDE is calculated from the mean CTDI_vol_ and the conversion factor, the middle of the scanning range, where ED is calculated, should be indicated in the depth image. There are two ways to register the *Z* position in the Kinect image. One is to simply put a small plastic or acrylic box with a certain thickness on the examination table before performing the Kinect acquisition. Since the thickness resolution of Kinect is 3.3 mm, objects larger than this thickness will produce changes in the grayscale of the depth image and appear in the edge enhanced image. Another method is to use the existing information on the examination table for positioning; see Figure 2(b). The edges of the table can be used as anchor points, which can be seen in the edge enhanced image (Figure 2(c)). This method does not need to add any physical markers and does not affect clinical workflow at all. Therefore, Kinect measurements can be easily integrated with these two SSDE correction methods.

The consumer grade somatosensory controller is commercially available and inexpensive. It has been widely used in medical applications recently. Researchers used Kinect to monitor the rehabilitation of limbs and bodies of patients with Parkinson's disease and after a stroke [17, 18]. Behrens et al. [19] investigated the applicability of Kinect to detect the walking speed of multiple sclerosis patients. Other applications include the gait analysis [20], balance training [21, 22], and monitoring patient setup [23, 24] and dose delivery during radiotherapy [25]. This study is the first to propose the use of Kinect on ED measurements and verify the results through clinical data.

In recent years, the use of CT scans in the pediatric population has increased markedly because no anesthesia is required under the high rotation speed of the CT gantry. Unnecessary and extensive scanning causes the sharp increase in medical exposure. However, children are more radiosensitive than adults [26]; the cancer risk of children is more than two times higher than that of adults [27], and the CT scan dose could also be more than twice that of adults [28], leading to dose estimates in pediatric CT more crucial than ever. Although using age as an index for ED estimates is simple, it may produce as much as 50% errors for children [12]. The major advantages of the proposed Kinect method are no radiation, accurate ED calculation, and automatic procedure. No manual measurements of LATD and APD are needed, which is time-saving for clinical practice. Additional radiation dose information can help radiologists to decide the necessity and justification of CT examination for pediatric patients in the view of radiation protection.

## 5. Conclusion

The Kinect somatosensory controller was integrated with the CT scanner to acquire depth images. The LATD and APD of subjects were measured automatically. The results obtained by Kinect matched those by *F* ruler and CT image very well. The proposed method has the advantages of simple, fast, low cost, no radiation, and automatic ED calculation. It can accurately measure LATD, APD, and ED in real time for size-specific dose estimates in CT.

## Figures and Tables

**Table 1:** Comparison of LATD, APD, and ED between *F* ruler and Kinect measurements.

	LATD (cm)	APD (cm)	ED (cm)
*F* ruler	28.99 ± 4.22	19.70 ± 3.78	23.88 ± 3.92
Kinect	29.14 ± 4.22	18.94 ± 5.83	23.46 ± 4.84
MAD	1.3%	5.2%	2.5%

**Table 2:** Simultaneous tests on *β*_0_ and *β*_1_ for Kinect versus *F* ruler.

	*H*_0_ : *β*_1_ = 1		*H*_0_ : *β*_0_ = 0	
	*T*	*p* value	*t*	*p* value
LATD	−0.455	0.651	0.012	0.991
APD	−0.906	0.367	2.115	0.037
ED	0.316	0.753	0.619	0.537

**Table 3:** Comparison of LATD, APD, and ED between CT and Kinect measurements.

	LATD (cm)	APD (cm)	ED (cm)
CT	30.71 ± 3.62	20.41 ± 3.50	25.01 ± 3.50
Kinect	30.49 ± 3.59	18.83 ± 5.26	23.93 ± 4.55
MAD	2.6%	8.8%	5.0%

**Table 4:** Simultaneous tests on *β*_0_ and *β*_1_ for Kinect versus CT.

	*H*_0_ : *β*_1_ = 1		*H*_0_ : *β*_0_ = 0	
	*t*	*p* value	*t*	*p* value
LATD	−0.679	0.506	0.542	0.595
APD	−0.469	0.644	−0.315	0.756
ED	−0.369	0.716	−0.293	0.773
